# A Case Report and Literature Review of Thyroid Storm Precipitated by COVID-19 Infection: A Crucial Pointer for Early Suspicion

**DOI:** 10.7759/cureus.35208

**Published:** 2023-02-20

**Authors:** Amulya Bellamkonda, Fareeza Mustafa, Tutul Chowdhury, Temesgen M Gobena, Renuka Bellamkonda

**Affiliations:** 1 Internal Medicine, One Brooklyn Health System, Brooklyn, USA; 2 Internal Medicine, Interfaith Medical Center, Brooklyn, USA; 3 Internal Medicine, Texas Health Methodist, Stephenville, USA

**Keywords:** thionamide, thyroid-storm, thyroid hormone disorders, graves´disease, coronavirus disease (covid-19), thyrotoxic crisis

## Abstract

Thyroid storm is a rare life-threatening condition characterized by severe and exaggerated clinical manifestations of thyrotoxicosis. It can be precipitated by a myriad of acute events and stressors including but not limited to surgery, trauma, or infections. Coronavirus disease 2019 (COVID-19) caused by the severe acute respiratory syndrome coronavirus 2 (SARS-CoV-2), primarily associated with respiratory symptoms, has been reported to be a likely precipitating cause of thyroid storm in a few cases. COVID-19 has been associated with both new-onset thyrotoxicosis and as a flare-up of the disease in remission. Even though the Burch-Wartofsky Point Scale (BWPS) scoring system has been used for years to help diagnose thyroid storms, the relatively low specificity of the score, especially in the setting of viral or bacterial infections, has been challenging for clinicians. Having a low threshold to consider the diagnosis of this life-threatening condition while at the same time meticulously ruling out other potential differential diagnoses is critical for saving lives. In this report, we discuss a case that highlights the importance that clinicians should accord to thyroid storm as one of the differential diagnoses in patients with a history of hyperthyroidism, with a positive test for COVID-19 infection on admission, and presenting with deranged vital signs and change in mentation from baseline.

## Introduction

Since its outbreak, coronavirus disease 2019 (COVID-19) caused by the severe acute respiratory syndrome coronavirus 2 (SARS-CoV-2), known for primarily causing respiratory symptoms, has been associated with multiple autoimmune and endocrine problems including thyroid disease. Angiotensin-converting enzyme (ACE2) combined with the transmembrane serine protease 2 (TMPRSS2), the key molecular complex used by SARS-CoV-2 as a target receptor to enter the tissue, has been found to be highly expressed in the thyroid gland as it is in the lungs [1,2]. In addition, due to the molecular mimicry between the proteins of SARS-CoV-2 with those in the thyroid, antibodies against SARS-CoV-2 were shown to react with the thyroid tissue [2] Therefore, either through the indirect effect of abnormal systemic inflammatory-immune responses caused by SARS-CoV-2 infection or through direct viral effect, SARS-CoV-2 may aggravate existing thyroid diseases or cause new abnormalities [2,3]. Even though there have been some reports of patients with Graves' disease (GD) following COVID-19 infection, particularly in patients with a previous history of thyroid diseases, only a few cases of thyroid storm during or following COVID-19 infection have been reported. In this report, we present a case with thyroid storm as the sole presenting feature of newly diagnosed COVID-19 infection in an elderly male patient with a previous history of thyroid disease.

## Case presentation

A 63-year-old male was transferred from the psychiatric floor to ICU after he was found to be in altered mental status associated with anxiety, agitation, fever, and chills of one-day duration. The patient had a past medical history of alcohol and polysubstance use disorder, iron deficiency anemia, paranoid schizophrenia, dementia, and hyperthyroidism. He was sweating profusely, had palpitations, and was very restless. The patient had been diagnosed with hyperthyroidism about three years prior and had been on methimazole and propranolol. His heart rate was 150 bpm, the temperature was 38.8 °C, respiratory rate was 20 bpm, BP was 134/71 mmHg, and oxygen saturation was 95% on room air. The patient was uncooperative for a detailed neurologic examination, but the rest of the physical examinations were normal. He tested positive for COVID-19 infection. On admission, mild normocytic normochromic anemia was detected on the complete blood count and the white cell count was within normal limits. Liver enzymes, renal function tests, and serum electrolytes were normal. Thyroid studies were abnormal (Tables 1, 2).

**Table 1:** Lab work on admission WBC: white blood cell; Hb: hemoglobin; MCV: mean corpuscular volume; BUN: blood urea nitrogen; eGFR: estimated glomerular filtration rate; Na: sodium; K: potassium; CO2: carbon dioxide; ALT: alanine transaminase; AST: aspartate aminotransferase; ALP: alkaline phosphatase; Ca: calcium; PT: prothrombin time; INR: international normalized ratio; PTT: partial thromboplastin time; LDH: lactate dehydrogenase; CRP: c-reactive protein; HBsAg: hepatitis B surface antigen; IgM: immunoglobulin M

Test	Reference range and units	Patient values
WBC	4.5-11.0 x 10^3^/uL	6 x 10^3^/uL
Hb	11.0-15.0 g/dL	12.2 g/dl
MCV	80-100 fL	84 fl
Platelets	130-400 x 10^3^/uL	225 x 10^3^/uL
BUN	7.0-18.7 mg/dL	19 mg/dl
Creatinine	0.57-1.11 mg/dL	0.5 mg/dl
eGFR	≥90.0	114.6
Na	136-145 mmol/L	142 mmol/L
K	3.5-5.1 mmol/L	3.3 mmol/L
CO_2_	22-29 mmol/L	27 mmol/L
Anion gap	4-12 mmol/L	10 mmol/L
Total bilirubin	0.2-1.2 mg/dL	0.4 mg/dl
ALT	10-55 U/L	50 U/L
AST	5-34 U/L	36 U/L
ALP	40-150 U/L	102 U/L
Albumin	3.5-5.2 g/dL	3.3 g/dL
Phosphorous	2.3-4.7 mg/dl	3.1 mg/dl
Ca	8.4-10.2 mg/dL	8.8 mg/dl
Lactate	0.50-1.90 mmol/L	1 mmol/L
PT	9.8-13.4 sec	13.9 sec
INR	0.85-1.15	1.21
PTT	24.9-35.9 sec	37.7 sec
Creatine kinase total	30.0-200.0 U/L	438 U/L
Vitamin D 25 hydroxy	30-100 ng/ml	12.8 ng/ml
Hemoglobin A1c	4.8-5.6%	5.8%
LDH	125-220 U/L	214 u/L
CRP	0.5-1 mg/dl	41 mg/dl
HBsAg	Negative	Negative
Hepatitis B core IgM	Negative	Negative
Hepatitis C	Negative	Negative

**Table 2:** Thyroid studies on admission TSH: thyroid-stimulating hormone; TSI: thyroid-stimulating immunoglobulin

Test	Reference range and units	Patient values
TSH	0.450-4.500 mIU/ml	<0.005
Free T4	0.82-1.77 ng/dl	3.85
T3	2.0-4.4 pg/ml	17.4
TSI	0.00-0.55 IU/L	0.86
Thyroid peroxidase antibody	0-34 IU/ml	390

**Figure 1:**
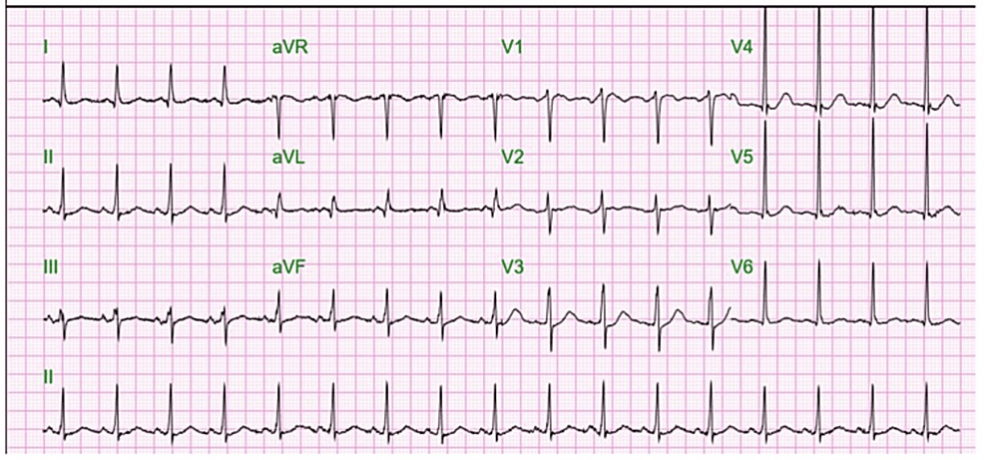
EKG on admission showing sinus tachycardia EKG: electrocardiogram

**Figure 2:**
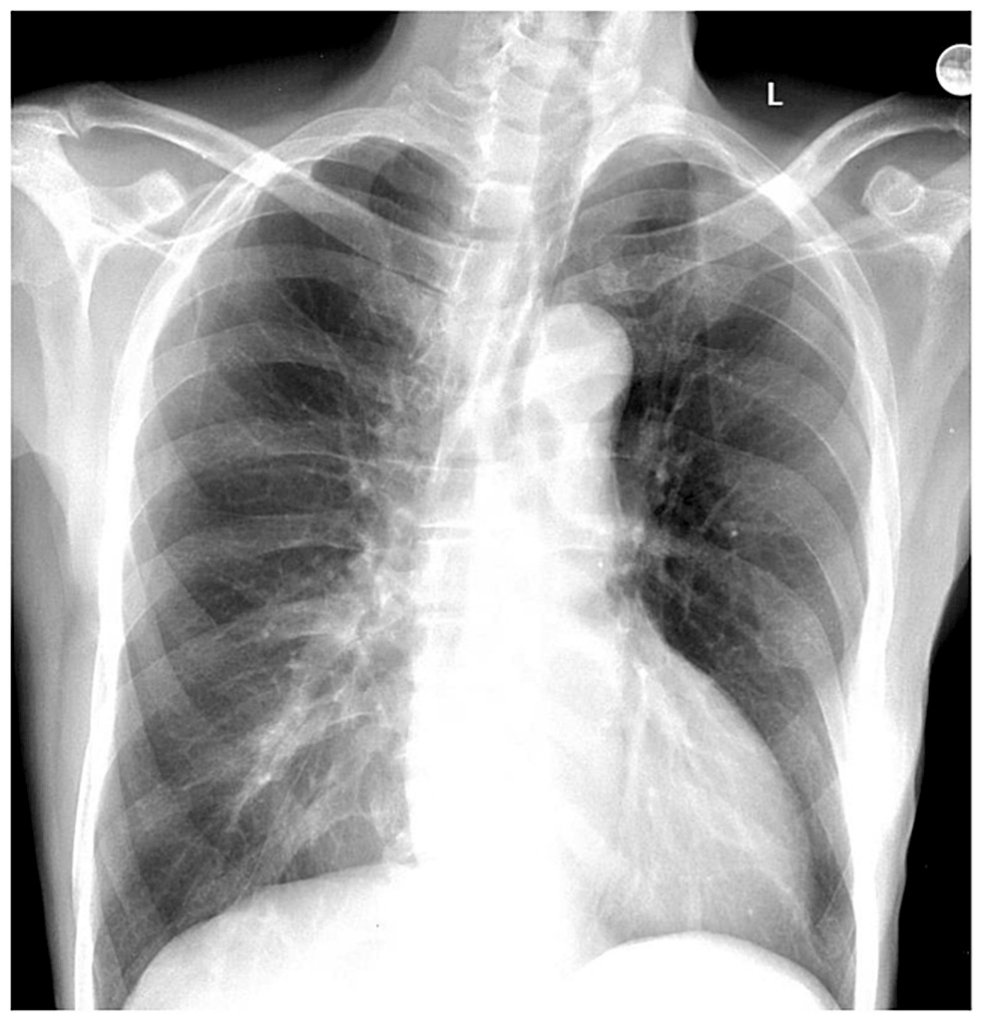
Chest X-ray showing normal appearance

EKG showed sinus tachycardia (Figure 1). Chest X-ray on admission showed very mild increased interstitial marking through the lung fields but no focal consolidation was noted (Figure 2).

**Figure 3:**
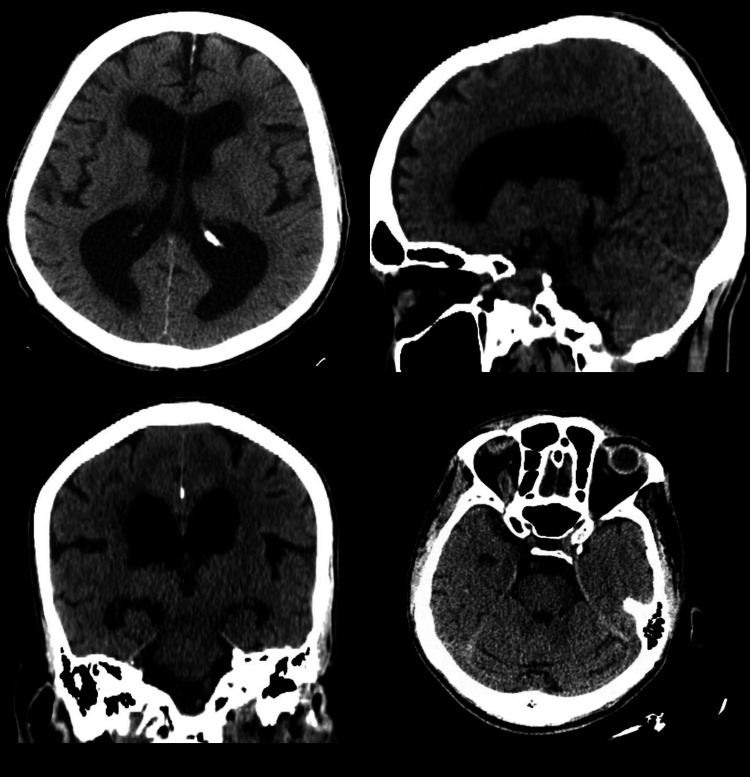
CT scan of the head showing no acute intracranial pathology CT: computed tomography

Non-contrast CT scan of the head was performed, which illustrated moderate diffuse cerebral atrophy without any evidence of intracranial mass, hemorrhage, infarct, hydrocephalus, skull fracture, or extra-axial fluid collection (Figure 3).

The patient was started on empiric antibiotics with pending blood culture results. According to the Burch-Wartofsky Point Scale (BWPS), our patient scored 65 points, highly suggestive of a thyroid storm. Endocrinology was consulted and treatment for thyroid storm was started with methimazole 20 mg PO every eight hours, propranolol 60 mg PO every six hours, hydrocortisone 100 mg PO every eight hours, and Lugol's iodine solution 10 drops orally three times a day, which was started six hours after the first dose of methimazole. The patient was also started on nirmatrelvir and ritonavir to treat COVID-19 infection. About 24 hours after admission to the ICU, mentation started to improve, and fever and tachycardia resolved. The patient continued to receive IV antibiotics, which were discontinued after the blood culture came back negative. Thyroid function results came back with low thyroid-stimulating hormone (TSH) (<0.015), elevated free T4 (2.29), and normal T3 (164). The thyroid-stimulating immunoglobulin (TSI) level was 0.86. After five days following treatment for thyroid storm, the patient was found to be stable with mentation at baseline. Hydrocortisone and Lugol's iodine were discontinued, and methimazole and propranolol doses were reduced. The patient was downgraded to the COVID-19 isolation floor.

**Figure 4:**
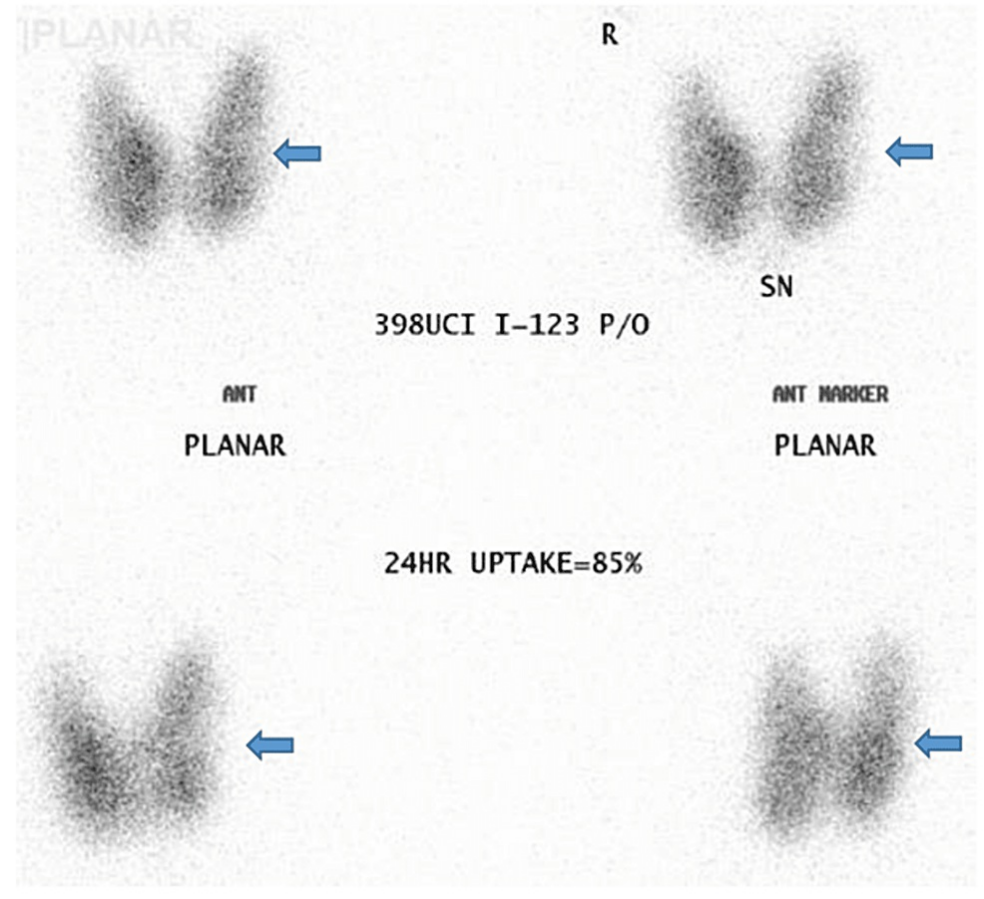
Radioactive iodine uptake revealed increased uptake suggestive of thyrotoxic crisis (blue arrows)

After the patient was consistently found to be stable with the new adjustment in the treatment regimen, methimazole was held for five days in preparation for a radioiodine uptake (RIU) scan to confirm the underlying thyroid disease. In RIU, there was a homogeneously increased radiotracer uptake noted throughout the thyroid lobes. A bolus thyroid lobe appeared to be enlarged (the right slightly more than the left) and the shape and configuration were normal. There were no extrinsic mass effects or shift of the thyroid lobes noted. The 24-hour thyroid uptake was calculated at 85% and homogeneously increased radiotracer uptake throughout the thyroid lobes (Figure 4). The thyroid lobes also appeared to be enlarged, which suggested the possibility of toxic goiter.

The patient was discharged with methimazole 20 mg oral daily and propranolol 10 mg three times daily. He was advised to follow up at the endocrine clinic within four to eight weeks.

## Discussion

The ongoing COVID-19 outbreak has been one of the major global pandemics in recent history. As per WHO, as of 2/1/2023, the total number of confirmed COVID-19 cases amounts to 753,651,712 globally, including 6,813,845 deaths [1]. It is now established that COVID-19 is a multisystemic disease.

It's long known that viral infections in general can trigger thyroid dysfunction resulting in subacute thyroiditis (SAT), nonthyroidal illness syndrome (NTIS), and autoimmune thyroid diseases (AITDs) including GD and Hashimoto’s thyroiditis. This report discussed a case of thyroid storm precipitated by SARS-CoV-2 infection in a patient with poorly controlled hyperthyroidism.

**Figure 5:**
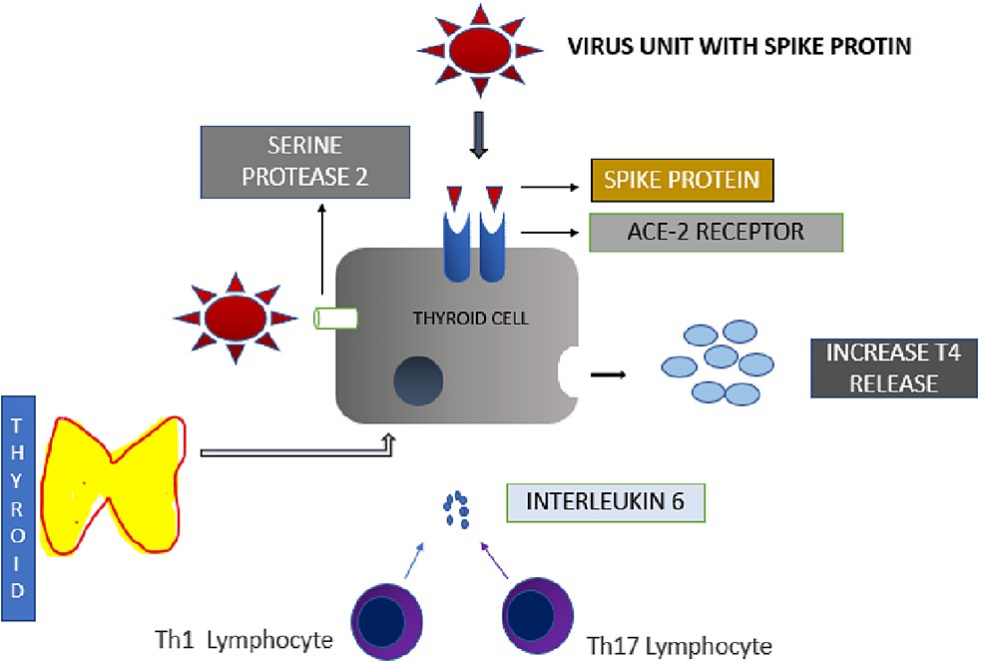
Graphical representation showing the mechanism of increased thyroid hormone release in COVID-19 infection* *[2] ACE-2: angiotensin-converting enzyme; COVID-19: coronavirus disease 2019

Multiple pathways have been implicated in the pathogenesis of thyrotoxicosis in the setting of SARS-CoV-2 infection. The ACE2 receptors are found extensively in all major organs and are also found to be expressed in high levels in thyroid tissue. The ACE2 receptors bind the spike proteins of CoV-2 viruses, aiding their entry into host cells [2]. In addition, the thyroid produces TMPRSS2, which enables virus entry [3]. Secondly, the abnormal immune hyperactivity involving Th1/Th17 lymphocytes leads to the release of inflammatory cytokines, progressing towards cytokine storm and causing thyroid dysfunction. IL-6 specifically causes thyroiditis with the release of preformed thyroid hormone from the gland [4,5]. The THYRCOV study [6] evaluated the thyroid function tests and serum IL-6, which were shown to be inversely related. This study showed that thyrotoxicosis was significantly associated with higher serum IL-6 levels. In addition, T3 and T4 have an effect on chemokine gene expression triggering the inflammatory processes, a hallmark of systemic viral infections [7,8]. A study by Wei et al. [8] showed the presence of apoptosis of follicular epithelium confirmed by TUNEL assay. Figure 5 illustrates the mechanism of increased thyroid hormone release in COVID-19 infection.

There is a considerable overlap of symptom presentation between thyroid storm and SARS-CoV-2 infection. A thorough history and timing of symptoms may be helpful in delineating the disease course. Fever, tachycardia, and atrial fibrillation are common in both conditions. Some patients may present with neck pain, which should be evaluated further to differentiate it from sore throat seen in COVID-19 infection. Lab data and clinical correlation are needed. It is not common to see thyroid storm as the sole clinical presentation of COVID-19 infection in the absence of other system involvement as occurred in this case.

**Figure 6:**
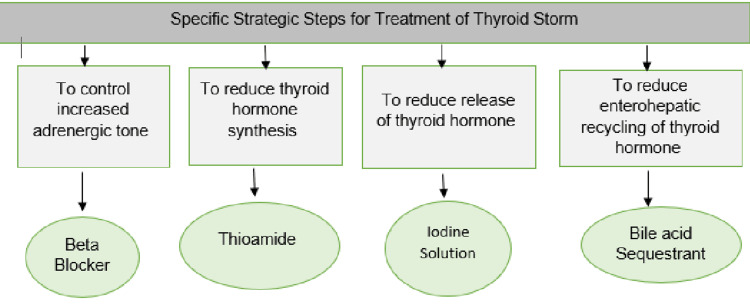
Strategic steps for the treatment of thyroid storm* *[6]

There is a lack of data regarding the specific treatment of thyroid storms in hospitalized cases of COVID-19. Therefore, expectant management with antithyroid drugs, beta-blockers, steroids, and supportive measures is recommended. The American Thyroid Association [8,9] recommends the use of propylthiouracil over methimazole, since it halts the T4 to T3 conversion in peripheral tissue, leading to faster clinical improvement. Hydrocortisone, beta-blockers, and inorganic iodine also reduce the T4 to T3 conversion. Figure 6 presents a graphical representation of the strategic steps for the treatment of thyroid storm.

## Conclusions

Most patients infected with the COVID-19 virus tend to present with a wide array of complications. We discussed a patient who presented with a thyroid storm. Caution is recommended in patients with a previous history of GD, as SARS-CoV-2 could provoke a thyrotoxic crisis as occurred in the case we report. A thyroid storm is considered a rare, life-threatening endocrine emergency. Early recognition of thyroid storm, in part with prompt COVID-19 testing, can help initiate therapy in a prompt manner and reduce mortality in these patients.

## References

[1] (2023). WHO Coronavirus (COVID-19) Dashboard. https://covid19.who.int/.

[2] Li MY, Li L, Zhang Y, Wang XS (2020). Expression of the SARS-CoV-2 cell receptor gene ACE2 in a wide variety of human tissues. Infect Dis Poverty.

[3] Clarke SA, Abbara A, Dhillo WS (2022). Impact of COVID-19 on the endocrine system: a mini-review. Endocrinology.

[4] Community Community, T.E. (2021 (2023). Elsevier Connect: Novel Coronavirus Information Center. https://www.elsevier.com/connect/coronavirus-information-center.

[5] Chen M, Zhou W, Xu W (2021). Thyroid function analysis in 50 patients with COVID-19: a retrospective study. Thyroid.

[6] Lania A, Sandri MT, Cellini M, Mirani M, Lavezzi E, Mazziotti G (2020). Thyrotoxicosis in patients with COVID-19: the THYRCOV study. Eur J Endocrinol.

[7] Scappaticcio L, Pitoia F, Esposito K, Piccardo A, Trimboli P (2021). Impact of COVID-19 on the thyroid gland: an update. Rev Endocr Metab Disord.

[8] Wei L, Sun S, Xu CH (2007). Pathology of the thyroid in severe acute respiratory syndrome. Hum Pathol.

[9] Ross DS, Burch HB, Cooper DS (2016). 2016 American Thyroid Association Guidelines for diagnosis and management of hyperthyroidism and other causes of thyrotoxicosis. Thyroid.

